# In vitro genome editing rescues parkinsonism phenotypes in induced pluripotent stem cells-derived dopaminergic neurons carrying *LRRK2* p.G2019S mutation

**DOI:** 10.1186/s13287-021-02585-2

**Published:** 2021-09-22

**Authors:** Kuo-Hsuan Chang, Cheng-Yen Huang, Chih-Hsin Ou-Yang, Chang-Han Ho, Han-Yi Lin, Chia-Lang Hsu, You-Tzung Chen, Yu-Chi Chou, Yi-Jing Chen, Ying Chen, Jia-Li Lin, Ji-Kuan Wang, Pei-Wen Lin, Ying-Ru Lin, Miao-Hsia Lin, Chi-Kang Tseng, Chin-Hsien Lin

**Affiliations:** 1 Department of Neurology, Chang Gung Memorial Hospital-Linkou Medical Center, Chang Gung University School of Medicine, Taoyuan, Taiwan; 2 The First Core Laboratory, College of Medicine, National Taiwan University, Taipei, Taiwan; 3 Department of Neurology, National Taiwan University Hospital and School of Medicine, Taipei, 100 Taiwan; 4 Department of Medical Research, National Taiwan University Hospital, Taipei, Taiwan; 5 Graduate Institute of Medical Genomics and Proteomics, College of Medicine, National Taiwan University, Taipei, Taiwan; 6 Biomedical Translation Research Center, Academia Sinica, Taipei, Taiwan; 7 Department of Microbiology, College of Medicine, National Taiwan University, Taipei, Taiwan

**Keywords:** Parkinson’s disease, *LRRK2*, Induced pluripotent stem cells, CRISPR-Cas9, Genome editing, Base editing

## Abstract

**Background:**

The c.G6055A (p.G2019S) mutation in leucine-rich repeat kinase 2 (*LRRK2*) is the most prevalent genetic cause of Parkinson’s disease (PD). CRISPR/Cas9-mediated genome editing by homology-directed repair (HDR) has been applied to correct the mutation but may create small insertions and deletions (indels) due to double-strand DNA breaks. Adenine base editors (ABEs) could convert targeted A·T to G·C in genomic DNA without double-strand breaks. However, the correction efficiency of ABE in *LRRK2* c.G6055A (p.G2019S) mutation remains unknown yet. This study aimed to compare the mutation correction efficiencies and off-target effects between HDR and ABEs in induced pluripotent stem cells (iPSCs) carrying *LRRK2* c.G6055A (p.G2019S) mutation.

**Methods:**

A set of mutation-corrected isogenic lines by editing the *LRRK2* c.G6055A (p.G2019S) mutation in a PD patient-derived iPSC line using HDR or ABE were established. The mutation correction efficacies, off-target effects, and indels between HDR and ABE were compared. Comparative transcriptomic and proteomic analyses between the *LRRK2* p.G2019S iPSCs and isogenic control cells were performed to identify novel molecular targets involved in LRRK2-parkinsonism pathways.

**Results:**

ABE had a higher correction rate (13/53 clones, 24.5%) than HDR (3/47 clones, 6.4%). Twenty-seven HDR clones (57.4%), but no ABE clones, had deletions, though 14 ABE clones (26.4%) had off-target mutations. The corrected isogenic iPSC-derived dopaminergic neurons exhibited reduced LRRK2 kinase activity, decreased phospho-α-synuclein expression, and mitigated neurite shrinkage and apoptosis. Comparative transcriptomic and proteomic analysis identified different gene expression patterns in energy metabolism, protein degradation, and peroxisome proliferator-activated receptor pathways between the mutant and isogenic control cells.

**Conclusions:**

The results of this study envision that ABE could directly correct the pathogenic mutation in iPSCs for reversing disease-related phenotypes in neuropathology and exploring novel pathophysiological targets in PD.

## Introduction

Parkinson’s disease (PD) is a multifactorial neurodegenerative disorder characterized by progressive neuronal α-synuclein aggregation and loss of dopaminergic neurons in the substantia nigra [1]. The etiology of PD comes from an interplay between genetic and environmental risk factors. Mutations in the leucine-rich repeat kinase 2 (*LRRK2*) gene have emerged as one of the most important genetic causes of familial and sporadic PD [2]. Patients carrying *LRRK2* mutations phenotypically manifest as late-onset sporadic PD [3, 4]. The c.G6055A (p.G2019S) mutation in *LRRK2* is the most prevalent mutation in patients with PD [5]. Cumulative evidence indicates that the *LRRK2* p.G2019S mutation aberrantly increases the LRRK2 kinase activity with regard to both autophosphorylation and the phosphorylation of exogenous kinase substrates [6]. This abnormally increased kinase activity has been linked to several pathogenic mechanisms in PD, including α-synuclein homeostasis, impaired neurite morphogenesis, and neuronal apoptosis [7–10].

The dominant gain-of-function mutation in *LRRK2* is challenging for the treatment of parkinsonism by gene silencing or gene disruption strategies because LRRK2 plays pivotal roles in many regulatory pathways, including the immune system [11, 12]. Although LRRK2 small molecule kinase inhibitors have already completed a phase I clinical trial in healthy volunteers [13], issues related to the safety of chronic use of LRRK2 kinase inhibitors, especially in the kidneys and lungs, are still concerning [14].

Clustered regularly interspaced short palindromic repeat (CRISPR)/CRISPR-associated protein 9 (Cas9) genome editing has been applied to correct specific disease mutation in the cells of patients by homology-directed repair (HDR) [15], providing isogenic controls without biological variance arising from differences in the genetic background. Recently, the successful correction of *LRRK2* mutations using zinc-finger nuclease-mediated gene targeting has been employed to create isogenic control lines by removing point mutations in induced pluripotent stem cells (iPSCs) generated from the fibroblasts of patients with *LRRK2* mutations [16]. However, CRISPR/Cas9-HDR involves the generation a double-strand DNA break that is also resolved via nonhomologous end joining (NHEJ). NHEJ typically results in random integration or complex mixtures of small insertions and deletions (indels) [17]. The recent advances in adenine base editors (ABEs) could convert targeted A·T base pairs to G·C base pairs without double-strand DNA breaks or donor DNA templates and function well in post-mitotic nondividing cells, such as neurons [18, 19]. Because ABE-mediated correction does not create double-strand DNA breaks, it minimizes the formation of indels [18, 20]. This study aimed to compare the mutation correction efficiencies and off-target effects between ABE and HDR.

A set of isogenic iPSC lines by correcting the *LRRK2* c.G6055A (p.G2019S) mutation in a PD patient-derived iPSC line using CRISPR/Cas9-based HDR or ABEs were established. The mutation correction efficacies, off-target effects, and indels were compared between the two genome editing methods. Comparative transcriptomic and proteomic analyses between the parental *LRRK2* p.G2019S iPSCs and isogenic control cells identified novel candidates involved in LRRK2-parkinsonism pathways.

## Materials and methods

### Human iPSC culture

Human iPSCs carrying the heterozygous *LRRK2* c.G6055A (p.G2019S) mutation were derived from a male Caucasian patient with PD and purchased from NINDS Human Genetics DNA and Cell Line Repository (ND40019*C). Wild-type (WT) iPSCs were derived from the peripheral blood mononuclear cells of a healthy 58-year-old male volunteer using the CytoTune Sendai viral vector kit (Life Technologies, Carlsbad, CA, USA). The collection of clinical information and venous blood from the healthy volunteer was approved by the Institutional Review Board of Chang Gung Memorial Hospital (201800469B0) and National Taiwan University Hospital (201907114RINB). As the age and gender information, in combination with other identifiers, may compromise patient/participant anonymity. Informed consent was obtained from this healthy volunteer, and we also have obtained the consent for publication from the donor. The iPSCs were maintained on a feeder-free culture using StemFlex medium (Gibco, Thermo Fisher Scientific, Waltham, Massachusetts, USA) according to the manufacturer’s instructions and passaged with StemPro Accutase (A1110501, Thermo Fisher Scientific, Waltham, Massachusetts, USA) at a ratio of 1:3 every week.

### CRISPR/Cas9-HDR-mediated genome editing

**Fig. 1:**
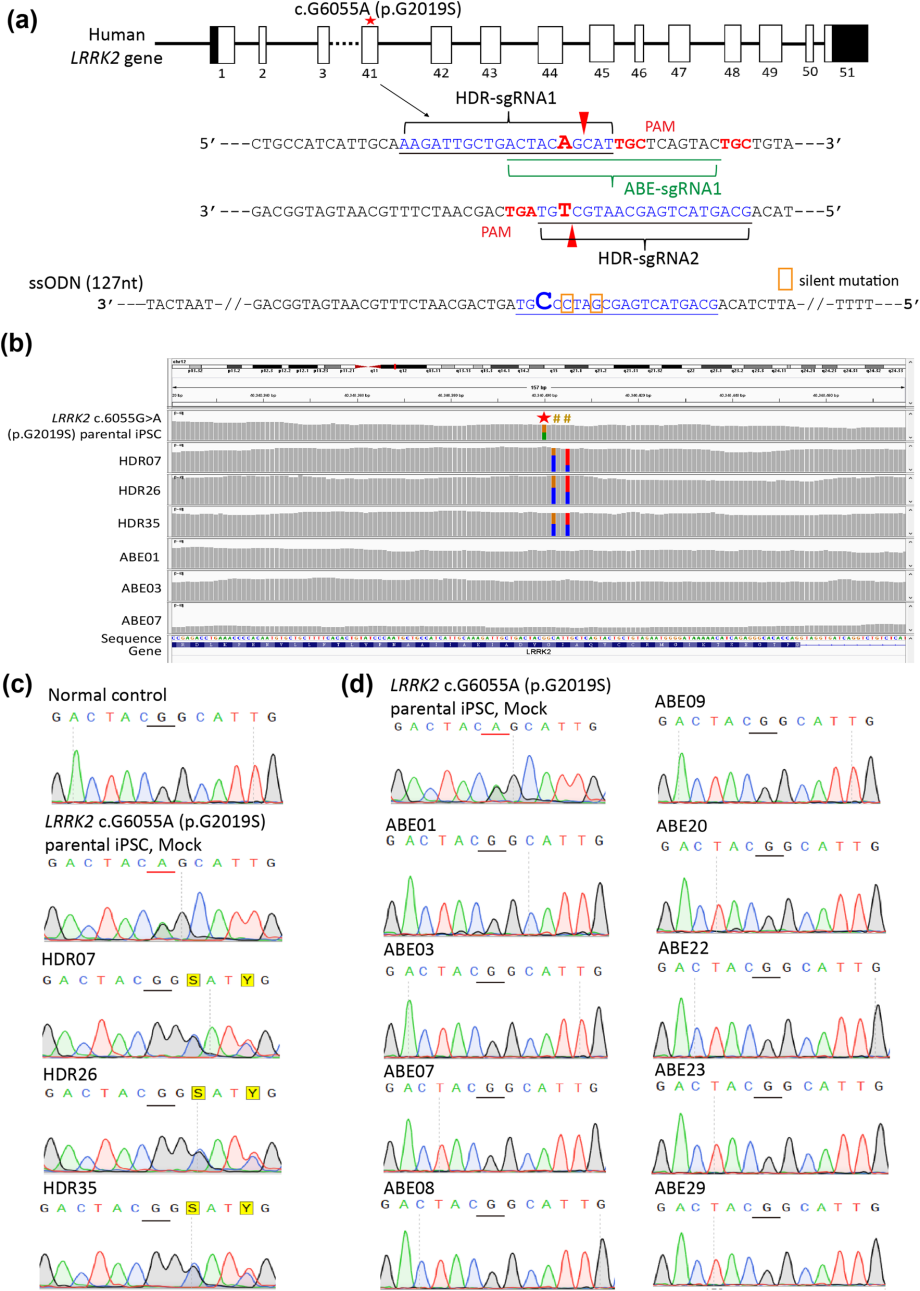
CRISPR/Cas9-HDR and ABE gene targeting strategies for correction of the *LRRK2* p.G2019S mutation in iPSCs from a PD patient. **a** Sequences of the sgRNAs for the HDR and ABE strategies and the ssODN donor template for HDR. Two silent mutations were introduced by the donor ssODN as cutting sites for the restriction enzyme. **b** Whole genome sequencing analysis of select clones revealed that the c.G6055A (p.G2019S) mutation in *LRRK2* was corrected to wild type and did not have indels or off-target editing in exon 41. The existence of the pre-designed silent variants as cutting sites for restriction enzymes in HDR-edited clones confirmed that the targeted region was replaced by HDR. **c** Sanger sequencing of exon 41 from the three isogenic iPSCs with mutations corrected by HDR. The designed silent variants confirmed that the targeted region was replaced by HDR. **d** Sanger sequencing of exon 41 from the isogenic iPSC clones with mutations corrected by ABE

To correct the *LRRK2* c.G6055A (p.G2019S) mutation and generate isogenic lines, double-nicking CRISPR/Cas9 and HDR were applied to the *LRRK2* p.G2019S iPSC line using the *Streptococcus pyogenes* (Sp)-derived Cas9 (SpCas9) system, which requires 5’-NGG as its protospacer-adjacent motif (PAM) sequence. A DNA double-strand break in the target site was induced as described previously [21]. The vectors were sequenced using a commercial sequencing kit (Applied Biosystems, Foster City, CA, USA) and a DNA sequencer (Applied Biosystems, Foster City, CA, USA) according to the manufacturer’s instructions. Several sgRNAs targeting c.G6055A (p.G2019S) in *LRRK2* (Fig. 1a) were designed. A puromycin/TK selection–counterselection cassette was inserted to facilitate screening and isolation of the corrected clones. The cleavage efficiency of the two designed sgRNAs was evaluated using a T7E1 cleavage assay after transfecting HEK293T cells with individual sgRNAs. The single-stranded oligodeoxynucleotides (ssODNs) used as donor templates harbored the WT sequence of exon 41 of *LRRK2* to replace the missense c.G6055A (p.G2019S) mutation via homologous recombination. The gRNAs and ssODNs were ordered from IDT (Coralville, USA).

### Adenosine base editing

The ABE system (ABEmax-NG and ABEmax-NGG) [22], supported by the RNAi core facility of Academia Sinica of Taiwan, was applied for direct correction of the c.G6055A (p.G2019S) mutation in *LRRK2*. The sequence of sgRNA targeting c.G6055A (p.G2019S) is shown in Fig. 1a.

### Electroporation of iPSCs

The iPSCs were grown to 70% confluence in a Matrigel (Corning)-coated 10-cm tissue culture dish. The cells were pre-treated with 10 μM of Rho Kinase (ROCK) inhibitor (Y-27632 dihydrochloride, Sigma-Aldrich, Burlington, USA) in 7.5 ml StemFlex™ medium (Thermo Fisher Scientific, Waltham, Massachusetts, USA) for 2 h and then washed with 5 ml Dulbecco's phosphate-buffered saline (DPBS) without Ca^2+^ and Mg^2+^. The cells were then dissociated into single cells by incubating them with 1 ml of StemPro Accutase for 5 min at 37 °C, and then mixing with 4 ml of StemFlex™ medium. The cells were then transferred to a 15 ml centrifuge tube and counted in a Neubauer-improved bright-line counting chamber (Marienfeld Superior, Lauda-Königshofen Germany). The 1 × 10^6^ cells were washed once with 1 ml DPBS, and then, the cell pellets resuspended in 100 μl Buffer R (Neon 100 μl kit, Thermo Fisher Scientific, Waltham, Massachusetts, USA). For CRISPR/Cas9-based HDR editing, resuspended cells were mixed with 5 μg of each sgRNA-encoding plasmid and 100 pmol of ssODN per electroporation (900 V/30 ms/2 pulses) in the Microporator MP-100 (Digital Bio Tech. Co., Korea). For ABE procedures, 1 × 10^6^ resuspended cells were mixed with 5 μg of ABEmax-NG plasmid and used for electroporation (1000 V/30 ms/2 pulses).

Next, cells were immediately seeded into a Matrigel-coated 6-well plate with 2 ml StemFlex™ medium containing 10 μM Y-27632. For cells receiving CRISPR/Cas9-based HDR editing, cells were treated with NHEJ inhibitor, 1 μM Scr7 (Merck, Darmstadt, Germany) [23], and HDR enhancer 10 μM RS-1 (Merck, Darmstadt, Germany) [24] to improve the efficacy of precise gene editing. After a 24-h incubation, the cells were treated with 2 ml StemFlex™ Medium containing 0.28 μg puromycin per ml medium (Merck, Darmstadt, Germany) with 1 μM Scr7 or 10 μM RS-1 for another 48 h. After puromycin treatment, half of the cells were harvested for evaluation of the HDR efficiency, and the other half were seeded on Matrigel-coated 96-well plates for single cell screening. All cell culture work was done at 37 °C in a humidified atmosphere containing 5% CO_2_.

### Off-target effect and whole genome sequencing analyses

The corrected clones obtained by the two gene editing methods were expanded and checked for off-target effects via direct Sanger sequencing of *LRRK2* exon 41. The off-target sites were also predicted at http://crispr.genome-engineering.org/. Whole genome sequencing (WGS) in selected clones was performed for validation. Raw reads were subjected to quality control analysis using FastQC (v0.11.9) and adaptor sequences trimmed by cutadapt (v3.0). Quality-controlled reads were mapped to human reference genome GRCh38 using BWA-mem (v0.7.17). According to the Genome Analysis ToolKit (GATK) Best Practices recommendations, duplicated reads were marked and base quality scores recalibrated using the functions implemented in GATK (v4.1.9.0). Variants were called utilizing GATK Mutect2 by comparing mutant and parental lines. Variants were annotated by AnnoVar (v2019-10-24) and Ingenuity Variant Analysis™. The quality of the alignment and variant calling was assessed by Integrative Genomics Viewer (IGV, v. 2.8.4).

### LRRK2 genotyping and sequencing

Genomic DNA was isolated from cells using a DNA Extraction Kit (Stratagene, La Jolla, CA, USA). The *LRRK2* genotype was determined by PCR amplification, gel purification, and direct sequencing using an ABI PRISM 3730 Genetic Analyzer (Applied Biosystems, Waltham, Massachusetts, USA). Each PCR included 20 ng of genomic DNA, 0.2 µM of each primer (Forward: TTAAGGGACAAAGTGAGCAC; Reverse: TGAACTCACATCTGAGGTCA), and Universal PCR Master Mix (Applied Biosystems, Waltham, Massachusetts, USA). The PCR conditions were as follows: 95 °C for 2 min, followed by 30 cycles of 95 °C for 30 s, 65 °C for 45 s, and 72 °C for 40 s, with a final extension at 72 °C for 10 min.

### Karyotyping

Confluent cells in 6-well plates were treated with 10 µg/ml N-desacetyl-N-methylocolchicine (Gibco, Thermo Fisher Scientific, Waltham, Massachusetts, USA) for 20 min at 37 °C and then dissociated with 0.025% trypsin. The cell suspension was centrifuged at 1300 rpm for 5 min. After washing with PBS, the cells were resuspended in 1 ml PBS and 3 ml 37.5 mM KCl hypotonic solution for 30 min at 37 °C and then treated with 2 ml methanol/acetic acid (3:1, v/v) at room temperature for 5 min. The cell suspension was centrifuged at 1300 rpm for 5 min and the pellet washed twice with 5 ml methanol/acetic acid for 5 min at room temperature. Two to three drops of cell suspension were dropped onto a microscope slide, which was then air-dried and stained by immersion in fresh Giemsa stain (Sigma-Aldrich, Burlington, USA). The chromosome spread was photographed under a Leica DMRB epifluorescence microscope.

### Teratoma formation and histology

Nude mice were anesthetized with diethyl ether, and the dorsal flank was subcutaneously injected with 100 μl of a cell suspension of 1 × 10^7^ cells/ml iPSCs in PBS containing 1% fetal calf serum. Four weeks after the injection, tumors were surgically dissected from the mice, fixed in 4% formaldehyde, and embedded in paraffin. Sections were stained with hematoxylin and eosin.

### Monolayer dopaminergic neuron differentiation

Dopaminergic neuron differentiation from mutation-corrected iPSCs was performed from the modified dual-inhibition monolayer differentiation protocol as described previously [25]. The iPSC cultures were disaggregated using disperse (STEMCELL Technologies, Vancouver, British Columbia, Canada) for 12 min and plated on Matrigel-coated dishes in MEF conditioned iPSC medium. The dishes were spiked with 8 ng/ml of fibroblast growth factor 2 (PeproTech, Rocky Hill, NJ, USA) at a density of 10,000–25,000 cells/cm^2^. The initial differentiation conditions were N2B27 medium (1:1 mixture of DMEM/F12 supplemented with modified N2 and Neurobasal medium supplemented with B27, all purchased from Invitrogen, Thermo Fisher Scientific, Waltham, Massachusetts, USA) spiked with 10 µM TGF-β inhibitor SB431542 (Sigma-Aldrich, Burlington, USA), 200 nM dorsomorphin (Sigma-Aldrich, Burlington, USA), and 100 ng/ml Noggin (R&D Systems, Minneapolis, MN, USA) for 4 days. Dopaminergic neuronal patterning was initiated with the addition of 20 ng/ml BDNF (R&D Systems, Minneapolis, MN, USA), 0.2 mM ascorbic acid (Sigma-Aldrich, Burlington, USA), 1 μM purmorphamine (Sigma-Aldrich, Burlington, USA), and 100 ng/ml FGF8 (R&D Systems, Minneapolis, MN, USA) on days 9–12. DAergic neurons were matured by treatment with 20 ng/ml BDNF, 0.2 mM ascorbic acid, 10 ng/ml GDNF, and 1 ng/ml TGFb3 (all were purchased from R&D, R&D Systems, Minneapolis, MN, USA).

### Immunocytochemistry staining

Cells cultured on coverslips were washed with PBS and fixed with 4% paraformaldehyde for 15 min at room temperature. After washing twice with PBS and 0.1% Triton X-100 (Sigma-Aldrich, Burlington, USA) at 4 °C for 5 min, the cells were incubated in blocking solution containing PBS and 1% BSA (Sigma-Aldrich, Burlington, USA) for 20 min at room temperature for 1 h. Next, they were incubated in primary antibody at the appropriate dilution in blocking solution overnight at 4 °C. After washing three times with PBS for 10 min, the cells were incubated with the diluted secondary antibody in blocking solution in the dark for 30 min. The cells were then counterstained with 4’-6-diamidino-2-phenylindole (DAPI, 1:10,000, Molecular Probes, Eugene, Oregon, USA). The coverslips were picked up and mounted with mounting solution (DAKO, Agilent Technologies, Santa Clara, CA, USA) on microscopic slides and examined under a Leica TCS confocal microscope.

### Western blot analysis

Cells were lysed with lysis buffer. The protein concentration of the cell lysate was measured by DC protein assay (Bio-Rad Laboratories, Hercules, CA, USA). Samples were diluted with 1% SDS to 0.7 mg/ml. A 12.5 µl aliquot of each sample was loaded into the wells of a 4.3% SDS–polyacrylamide stacking gel and separated by electrophoresis on a 10% SDS–polyacrylamide running gel in running buffer at 120 V for 60–90 min. The gels were then placed in a semi-dry transfer cell (Bio-Rad Laboratories, Hercules, CA, USA) in which the polyvinylidene difluoride membrane (Merck Millipore, Burlington, Massachusetts, USA) had been rinsed in methanol. Proteins were transferred by running at 90 V for 1 h in transfer buffer. The membranes were blocked with 3% skim milk in PBS containing 0.05% Tween-20 (Sigma-Aldrich, Burlington, USA) for 1 h at room temperature and then incubated with primary antibody appropriately diluted with 5% skim milk in PBST at 4 °C overnight. After washing with PBST, the membrane was incubated with horseradish peroxidase (HRP)-conjugated secondary antibody for 1 h at room temperature. Signals were detected by enhanced chemiluminescence and Hyperfilm ECL (Bio-Rad Laboratories, Hercules, CA, USA).

### Antibodies

The primary antibodies used for immunocytochemistry were rabbit anti-OCT4 (1:500, Abcam), mouse anti-TRA-1-81 (1:250, BD Pharmingen), rabbit anti-TH (tyrosine hydroxylase, 1:250, Millipore), and mouse anti-TUBB3 (1:10,000, GeneTex). The secondary antibodies were Alexa594-conjugated donkey anti-rabbit IgG (1:200, Invitrogen) and Alexa488-conjugated donkey anti-mouse IgG (1:200, Invitrogen). The antibodies used for Western blotting were rabbit anti-α-synuclein (1:800, Proteintech), rabbit anti-phosphor-α-synuclein (Ser129) (1:1000, Abcam), rabbit anti-LRRK2 (1:1000, Abcam), rabbit anti-phospho-LRRK2 (Ser1292) (1:500, Abcam), and mouse anti-TUBB3 (1:10,000, Biolegend). The secondary antibodies were goat anti-mouse IgG-HRP (1:20,000, GE Healthcare) and goat anti-rabbit IgG-HRP (1:20,000, GE Healthcare).

### Caspase 3 activity assay

Caspase 3 activity was determined using a Caspase Activity Assay Kit (Sigma-Aldrich, Burlington, USA). The activity was measured according to the cleavage of DEVD-p-nitroanilide to yellow-colored p-nitroaniline through time [26]. The caspase 3 activity was calculated in µmol p-nitroaniline released per min per ml of cell lysate. All samples were tested as triplicates and normalized to normal control.

### Neurite outgrowth measurement

The neurite outgrowth features of differentiated neurons, including total outgrowth, processes, and branches, were assessed by MetaMorph microscopy automation and image analysis software (Molecular Devices, San Jose, CA, USA) based on the immunocytochemistry of DA neuronal marker TH.

### Transcriptomic analysis

#### RNA quantification and qualification

Cells were collected in Trizol reagent (Ambion, Life Technologies, Carlsbad, CA, USA) according to the manufacturer’s instructions and then treated with DNase I (New England Biolabs, Ipswich, Massachusetts, USA) at 37 °C for 30 min. Total RNA was extracted once using phenol/chloroform equilibrated with 50 mM NaOAc (pH 5.0) and ethanol precipitated. RNA quantification was performed using SimpliNano™ - Biochrom Spectrophotometers (Biochrom, MA, USA). RNA degradation and integrity were monitored by the Qsep 100 DNA/RNA Analyzer (BiOptic Inc., Taiwan).

#### Library preparation for transcriptome sequencing

A total of 1 µl of total RNA was used as the input for library preparation. Sequencing libraries were generated using the KAPA mRNA HyperPrep Kit (KAPA Biosystems, Roche, Basel, Switzerland) according to the manufacturer’s recommendations. Index codes were added to attribute sequences to each sample. Briefly, mRNA was purified from total RNA using magnetic oligo-dT beads. Captured mRNA was fragmented by incubating at 94 °C in the presence of magnesium in KAPA Fragment in 1X Prime and Elute Buffer. First-strand cDNA was synthesized using random hexamers. Combined second-strand synthesis and A-tailing, which converts the cDNA/RNA hybrid to double-stranded cDNA, incorporated dUTP into the second cDNA strand and added dAMP to the 3′ ends of the resulting double-stranded cDNA. The dsDNA adapter with 3’dTMP overhangs was ligated to library insert fragments to generate the library fragments carrying the adapters. To select cDNA fragments 300 ~ 400 bp in length, the library fragments were purified using the KAPA Pure Beads system (KAPA Biosystems, Roche, Basel, Switzerland). The library carrying appropriate adapter sequences at both ends was amplified using KAPA HiFi HotStart Ready Mix (KAPA Biosystems, Roche, Basel, Switzerland) with library amplification primers. The strand marked with dUTP was not amplified, allowing strand-specific sequencing. PCR products were purified using the KAPA Pure Beads system and the library quality assessed on the Qsep 100 DNA/RNA Analyzer (BiOptic Inc., Taiwan).

### Transcriptome data analysis

The raw data obtained by high-throughput sequencing (Illumina NovaSeq 6000 platform) were transformed into raw sequenced reads by CASAVA base calling and stored in the FASTQ format. FastQC and MultiQC [27] were used to estimate the quality of the files. The paired-end reads were filtered by Trimmomatic (v0.38) [28] to discard low-quality reads, trim adaptor sequences, and eliminate poor-quality bases with the following parameters: LEADING:3 TRAILING:3 SLIDINGWINDOW:4:15 MINLEN:30. The obtained high-quality data (clean reads) were used for subsequent analysis. Read pairs from each sample were aligned to the reference genome (e.g., H. sapiens, GRCh38) by HISAT2 software (v2.1.0) [29, 30]. The read numbers mapped to individual genes were counted by Feature Counts (v1.6.0) [31]. For gene expression, “Trimmed Mean of M-values” normalization (TMM) was performed by edgeR (v3.28.1) based on negative binomial without biological replicates. Differentially expressed genes were analyzed in R using DEGseq (v1.40.0), which is based on a Poisson distribution model. The resulting p-values were adjusted using Benjamini and Hochberg’s approach for controlling the false discovery rate (FDR). For transcripts per million (TPM), the read counts were normalized by the gene length (per kilobase) and then divided by the sum of the gene length normalized values and multiplied by 1 × 10^6^.

### Proteomic analysis

#### Protein extraction and digestion

Cell pellets were washed three times with ice-cold PBS and resuspended in lysis buffer containing 4% SDS, 1X protease inhibitor cocktail (BIOTOOLS, Taipei, Taiwan), and 100 mM Tris–HCl (pH 9.0). First, cells were heated at 95 °C for 5 min to inactivate the endogenous enzymes and then subjected to ultrasonication for homogenization. Homogenized samples were centrifuged for 30 min at 20,000 rpm at 4 °C and the supernatants subjected to methanol/chloroform precipitation [32]. The precipitated protein extract was dissolved and denatured in 8 M urea buffer containing 50 mM TEABC, 10 mM TCEP, and 40 mM CAA. After dilution to 2 M urea with 50 mM TEABC, lysyl endopeptidase was added at a 1:100 (w/w) ratio for 3 h, followed by overnight trypsin digestion at a ratio of 1:50 (w/w) at 29 °C. Digested samples were acidified with TFA to pH 2–3 and desalted using a homemade SDB-XC StageTip [33].

#### LC–MS/MS analysis

Tryptic peptides were analyzed using an Orbitrap Fusion Lumos mass spectrometer (Thermo Fisher Scientific, Waltham, Massachusetts, USA) coupled with a Thermo Scientific UltiMate 3000 RSLCnano system (Thermo Fisher Scientific, Waltham, Massachusetts, USA). One microgram of peptide mixture was loaded onto the Thermo Scientific PepMap C18 50 cm × 75 µm ID column (Thermo Fisher Scientific, Waltham, Massachusetts, USA) and separated using a gradient of 4% to 38.5% solvent B (ACN with 0.1% formic acid) over 160 min at a flow rate of 250 nl/min and a column temperature of 45 °C. Solvent A was 0.1% formic acid in water. The mass spectrometer was operated in TopSpeed mode with a cycle time of 3 s. Survey full-scan MS spectra were acquired in the orbitrap (m/z 350–1500) with the resolution set to 60 K and automatic gain control (AGC) target at 5e4. The most intense ions were isolated sequentially for HCD MS/MS fragmentation and detected in the orbitrap by dynamic exclusion for 20 s. For MS/MS, a resolution of 30 K and an isolation window of 1.4 Th with an auto-tuned AGC target and injection time were applied. Fragmentation was performed with a normalized collision energy of 30%. The advanced peak detection function was on with a precursor fit threshold of 70% at a 1.4 m/z window. The precursor ions with charges of 2^+^ to 7^+^ were selected for HCD fragmentation.

#### MS data processing

All MS raw files were processed in Proteome Discoverer (ver. 2.4) by SequestHT engines against the Swissprot Homo sapiens database (download on 2020.02) and common contaminants containing 20,303 and 147 protein entries for protein identification and quantification, respectively. The search criteria were as follows: 10 ppm mass tolerance for precursor and 0.05 Da for product ions, trypsin specificity allowing up to two missed cleavages, fixed modification of carbamidomethyl (C), and variable modification of oxidation (M) and acetylation (protein N-term). The minimal peptide length was set at seven residues, and the FDRs for peptide and protein were both set at 1%. Master proteins were selected for advanced quantification analysis using Perseus (ver. 1.6.14.0) [34]. To detect the complete matrix of intensities for regulation in the quantification analysis, missing value imputation was performed when all three values existed in at least one condition. The log2 scale of protein abundance was subjected to a Student’s t test with *P* < 0.05. All raw MS files are accessible at the jPOST repository (https://repository.jpostdb.org) under accession number JPST001139 [35].

### Data analysis

Quantification experiments were performed in triplicate. Neurite outgrowth measurements were obtained by counting at least 200 neurons in each independent experiment. Each set of values was expressed as mean ± standard deviation. Differences between groups were determined using a two sample Student’s *t* test or one-way ANOVA with Bonferroni’s post hoc test. All *P*-values were two-tailed, and a value less than 0.05 was considered significant. All statistical analyses were performed using the Statistical Program for Social Science software version 23.

## Results

### Comparison of CRISPR/Cas9-HDR and ABE genome editing

To correct the c.G6055A (p.G2019S) mutation in *LRRK2* and generate isogenic lines, CRISPR/Cas9-based HDR and ABEs were applied. Double-nicking CRISPR/Cas9-HDR was applied to the mutant iPSC line using a single-stranded oligodeoxynucleotides (ssODNs) template harboring the WT sequence of exon 41 of *LRRK2* and containing silent variants as cutting sites for restriction enzymes on the 5’ side of the target site to replace the targeted gene region via homologous recombination (Fig. 1a). The cleavage efficiency of the sgRNAs for CRISPR/Cas9-HDR was evaluated by a T7E1 cleavage assay after transfecting HEK293T cells with individual sgRNAs. Following electroporation of the CRISPR/Cas9-HDR plasmid and ssODNs into the *LRRK2* p.G2019S iPSCs, puromycin selection was performed and ∼50 colonies were isolated and evaluated by Sanger sequencing to confirm the targeted mutation was corrected. Among the 47 clones generated by HDR, 3 (6.4%) were on-targeted corrected, whereas the ABEs had a much higher correction rate (13 of 53 clones, 24.5%). Twenty-seven of the HDR clones (57.4%), but none of the ABE clones, had small deletions on exon 41 of *LRRK2*. However, the ABEs created 14 clones (26.4%) with off-target missense mutations (Table 1). The WGS analysis of clones corrected for the c.G6055A (p.G2019S) mutation in *LRRK2* did not reveal indels or off-target editing on exon 41, which confirmed that no off-target sites were located within known annotated genes or within genes homologous to the more highly annotated human genome (Fig. 1b). Sanger sequencing of these clones confirmed correction of the mutation in these clones using HDR (Fig. 1c) and ABEs (Fig. 1d). The existence of the pre-designed silent variants in HDR-edited clones confirmed that the targeted region was replaced (Fig. 1b, c). Among the successfully corrected clones without off-target effects or indels, one clone from each editing group, ABE01 and HDR26, was selected for further characterization of PD-related features.

**Table 1:** Comparison of the mutation correction efficacies, off-target, and indels rates between CRISPR/Cas9-HDR and ABEs

	HDR	ABE
Number of colonies	47	53
Insertions or deletions on exon 41	27 (57.4%), all are small deletions	0
Creating additional missense mutations	0	14 (26.4%): 11 (20.75%) are within active window 3 (5.67%) are proximal off-target editing
Genotype: still *LRRK2* c.6055G > A mutation without correction	17 (36.2%)	26 (49.1%)
Genotype: corrected (*LRRK2* c.6055A > G)	3 (6.4%)	13 (24.5%)

HDR, Homology-directed repair; ABE, adenine base editing

### Characterization of HDR- and ABE-corrected LRRK2 p.G2019S iPSCs

**Fig. 2:**
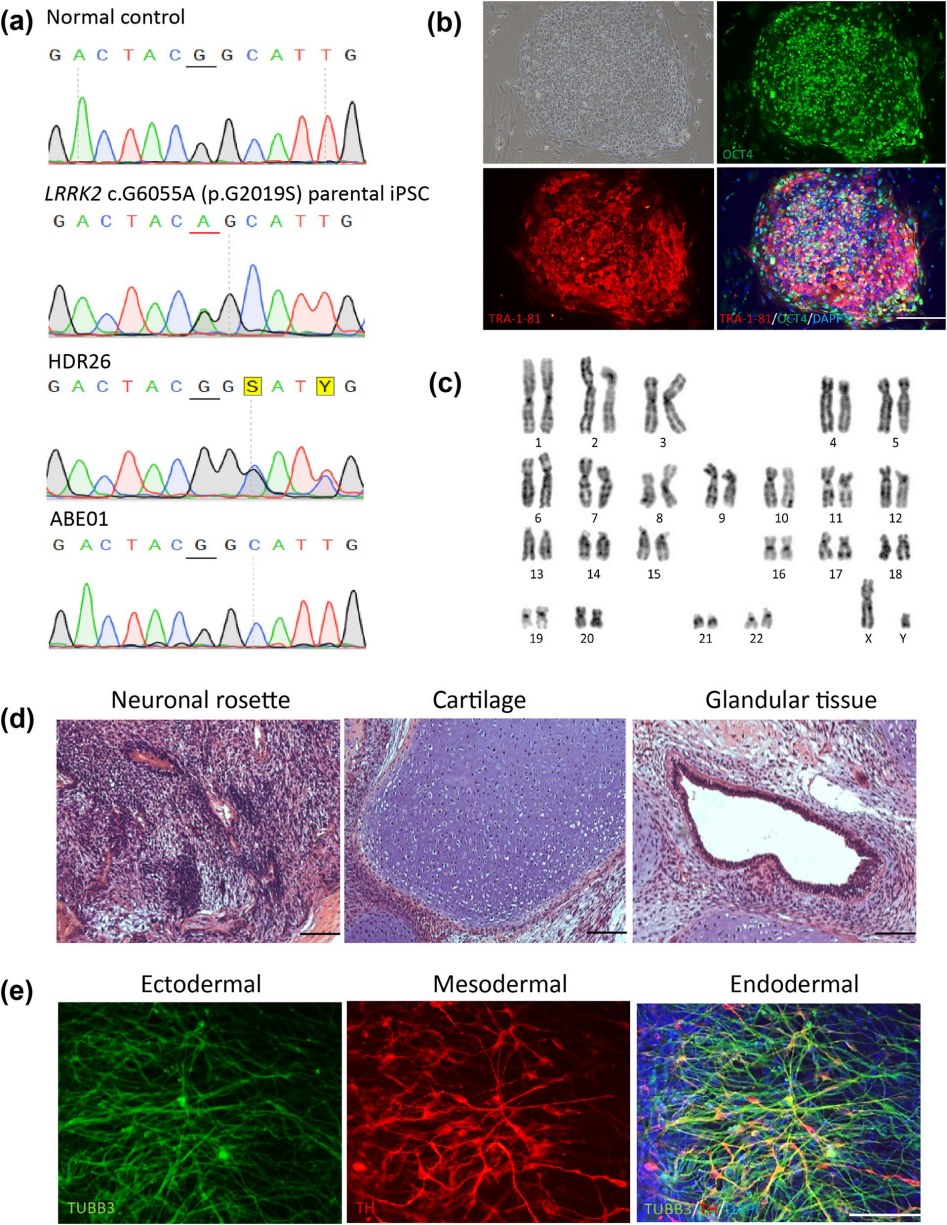
Characterization of induced pluripotent stem cells corrected by genomic editing. **a** DNA sequencing at position 6055 of exon 41 in *LRRK2*. HDR generated two silent mutations introduced by donor DNA. **b** Bright-field image and immunofluorescent staining for OCT4 (green) and TRA-1-81 (red) in corrected iPSCs (ABE01). Nuclei are shown by DAPI staining (blue). Scale bar = 100 µm. **c** Karyotype of ABE01. **d** Teratoma formed after subcutaneous injection of ABE01 into NUD mice. **e** DA neurons derived from ABE01 co-expressing TUBB3 (green) and TH (red). Nuclei are shown by DAPI staining (blue). Scale bar = 100 µm

**Fig. 3:**
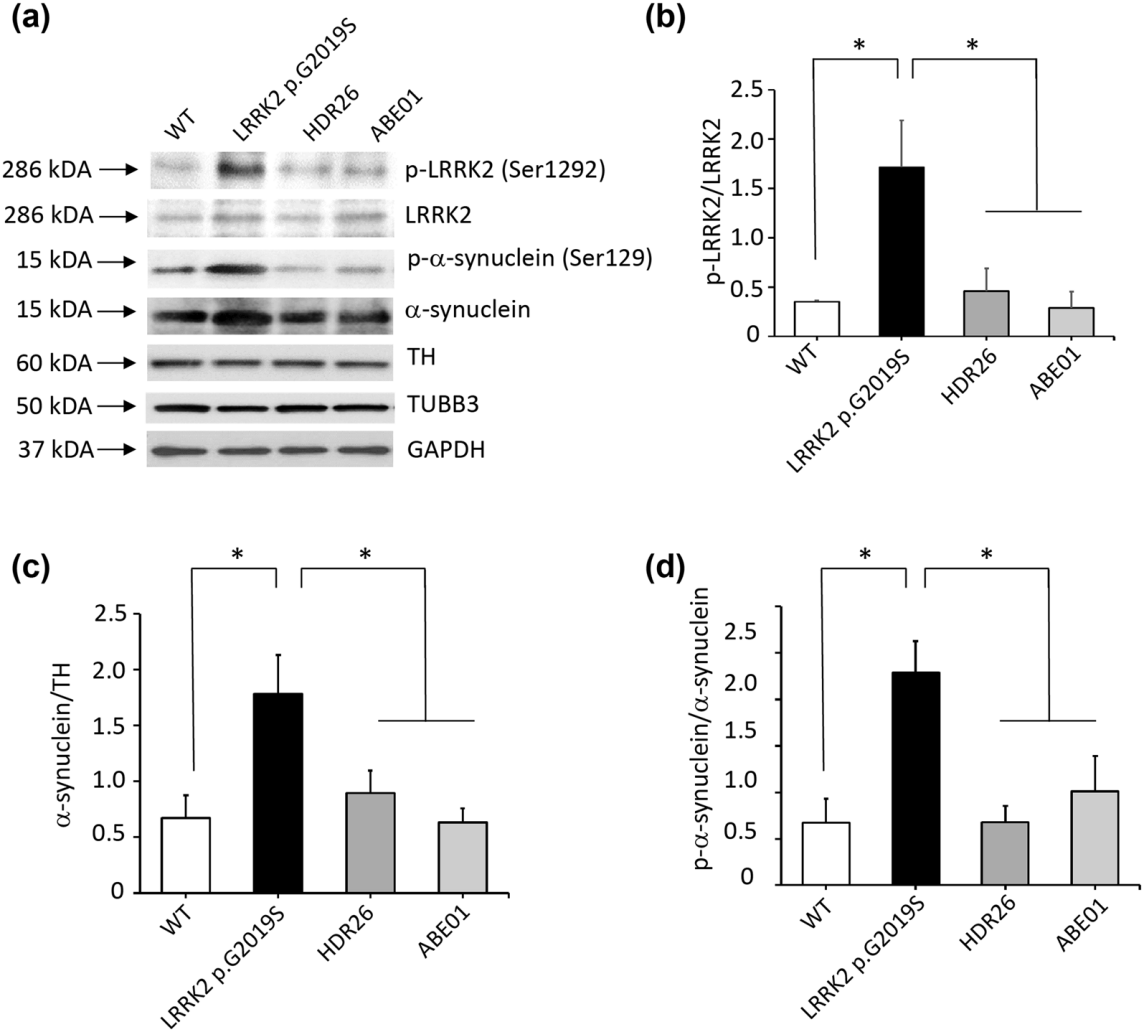
LRRK2 phosphorylation and α-synuclein accumulation in DA neurons derived from iPSCs. **a**, **b** Western blotting demonstrated reduced phosphorylation of Ser1292 in LRRK2 and **a**, **c** Ser129 in α-synuclein. **a**, **d** Increased levels of α-synuclein were also observed in iPSCs carrying the *LRRK2* p.G2019S mutation (G2019S) compared to wild-type iPSCs (WT). Isogenic clones (ABE01 and HDR26) with corrected mutations rescued these phenotypes for Parkinson’s disease. Western blotting was performed in triplicate. *P < 0.05 compared to WT, ABE01, and HDR26

To characterize successfully corrected clones, ABE01 and HDR26 were expanded in feeder-free culture. Both clones carried a homozygous wild-type G allele at position 6055 of exon 41 of *LRRK2* (Fig. 2a, upper panel). The HDR26 clone had two silent mutations that were introduced by the donor ssODN (Fig. 2a, lower panel). Both ABE01 and HDR26 iPSCs were successfully cultured in an undifferentiated state for more than 35 passages (10 months), formed dome-shaped and dense human embryonic stem cell-like colonies, and expressed the pluripotent stem cell marker OCT4 and TRA-1-81. (A representative image of HDR26 is shown in Fig. 2b.) These iPSC clones demonstrated normal chromosome karyotypes (Fig. 2c) and formed teratomas in nude mice (Fig. 2d). Using a feeder-free, chemically defined, in vitro differentiation protocol, these iPSC clones were differentiated into dopaminergic neurons. Cells expressing neuronal marker TUBB3 were generated following 12 weeks of differentiation, whereas 60–90% of the iPSC-derived neurons expressed the dopaminergic neuronal marker tyrosine hydroxylase (TH) (Fig. 2e, middle panel). The expression levels of TH and TUBB3 among neurons derived from different iPSCs were comparable (Fig. 3a), suggesting the identical efficiencies of neuronal differentiation in different iPSC clones.

### Correction of PD phenotypes

**Fig. 4:**
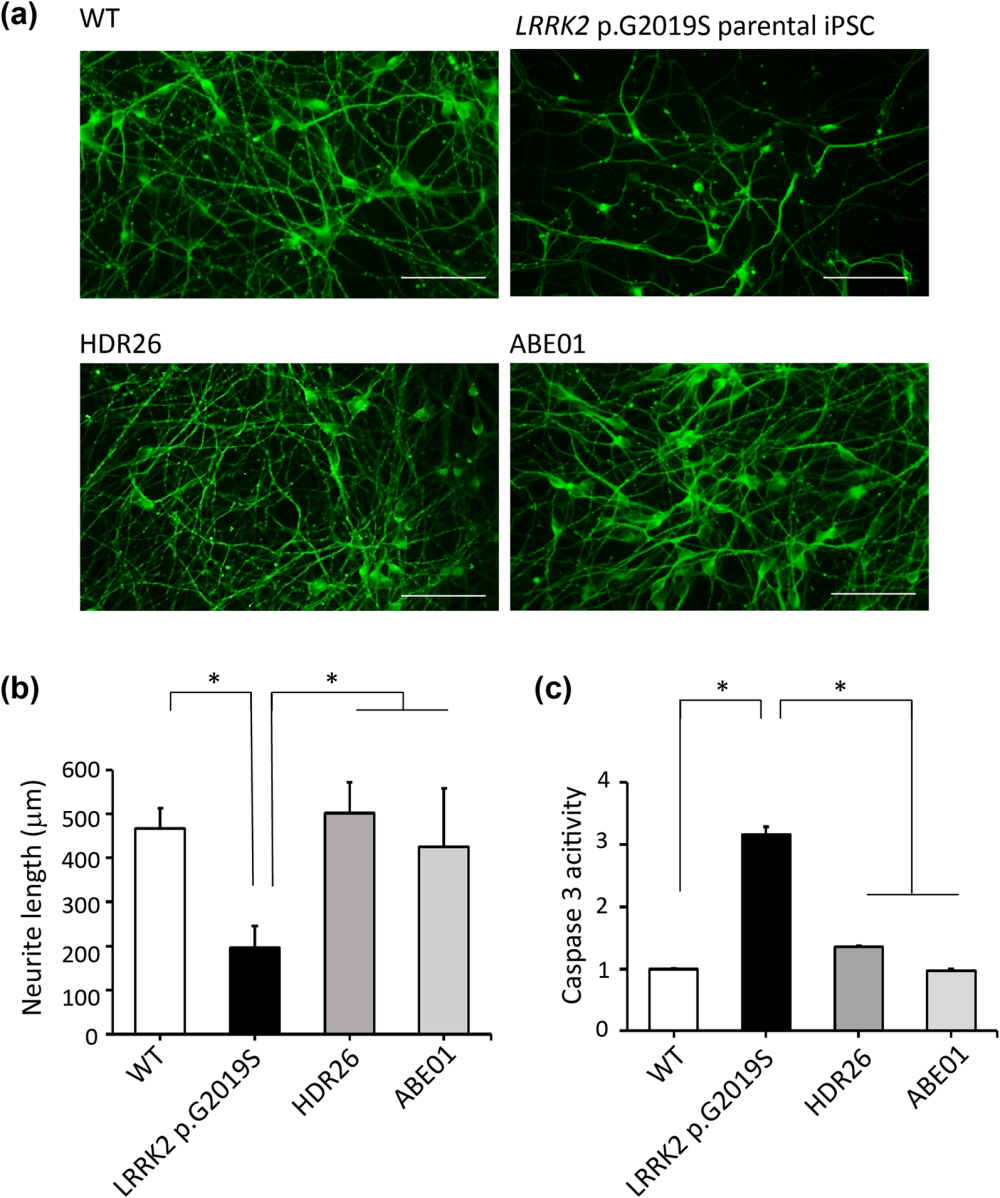
Neurite outgrowth and caspase 3 activities in DA neurons derived from iPSCs. **a**, **b** Reduced neurite outgrowth and **c** increased caspase 3 activities were observed in iPSCs carrying the *LRRK2* p.G2019S mutation (G2019S) compared to wild-type iPSCs (WT). Isogenic clones (ABE01 and HDR26) with mutations corrected rescued these phenotypes for Parkinson’s disease. Caspase 3 activities were measured in triplicate for each iPSC line and normalized to WT. *P < 0.05 compared to WT, ABE01, and HDR26

The *LRRK2* c.G6055A (p.G2019S) mutation significantly increases the kinase activity of LRRK2, leading to increased autophosphorylation, α-synuclein phosphorylation, and dopaminergic neuronal degeneration [6]. To determine whether correction of the *LRRK2* c.G6055A (p.G2019S) mutation by genome editing affected the LRRK2 kinase activity, the autophosphorylation site at Ser1292 of LRRK2 was examined [36]. Dopaminergic neurons derived from *LRRK2* p.G2019S iPSCs demonstrated elevated phosphorylation at Ser1292 (p-LRRK2/LRRK2: 1.71 ± 0.47), but this autophosphorylation was reduced in those derived from ABE01 (p-LRRK2/LRRK2: 0.29 ± 0.17, *P* = 0.005 compared to *LRRK2* p.G2019S) and HDR26 (p-LRRK2/LRRK2: 0.46 ± 0.23, *P* = 0.012 compared to *LRRK2* p.G2019S, Fig. 3a, b). The elevated expression of total α-synuclein in *LRRK2* G2019S iPSC-derived dopaminergic neurons (α-synuclein/TH: 1.78 ± 0.35) was also reduced in those derived from ABE01 (α-synuclein/TH: 0.63 ± 0.13, *P* = 0.007 compared to *LRRK2* p.G2019S) or HDR26 (α-synuclein/TH: 0.89 ± 0.20. *P* = 0.032 compared to *LRRK2* p.G2019S, Fig. 3a, c). The pathological phospho-α-synuclein at Ser129 [37] was also elevated in *LRRK2* p.G2019S-derived dopaminergic neurons (p-α-synuclein/α-synuclein: 0.64 ± 0.07), but this phosphorylation was largely reduced in ABE01 (p-α-synuclein/α-synuclein: 0.20 ± 0.08, *P* = 0.016 compared to *LRRK2* p.G2019S) and HDR26-derived dopaminergic neurons (p-α α -synuclein/α-synuclein: 0.14 ± 0.03, *P* = 0.004 compared to *LRRK2* p.G2019S, Fig. 3a, d). The impaired neurite outgrowth of *LRRK2* p.G2019S iPSC-derived dopaminergic neurons (196.95 ± 49.84 μm) was also restored in those derived from ABE01 (424.92 ± 132.42 μm, *P* < 0.001 compared to *LRRK2* p.G2019S) and HDR26 (500.60 ± 69.61 μm, *P* < 0.001 compared to *LRRK2* p.G2019S, Fig. 4a, b). The caspase 3 activity in *LRRK2* G2019S iPSC-derived dopaminergic neurons was elevated (fold change: 3.16 ± 0.12), supporting the previous findings that *LRRK2* c.G6055A (p.G2019S) mutation promotes neuronal apoptosis [7, 9]. The mutation-corrected ABE01 (fold change: 0.97 ± 0.02, *P* < 0.001 compared to *LRRK2* p.G2019S) and HDR26 iPSC-derived dopaminergic neurons (fold change: 1.35 ± 0.02, *P* < 0.001 compared to *LRRK2* p.G2019S) showed reduced caspase 3 activity (Fig. 4c). Thus, the pathological hallmarks of *LRRK2*-parkinsonism, including increased LRRK2 kinase activity, aberrant α-synuclein expression and phosphorylation, impaired neurite outgrowth, and increased apoptosis in dopaminergic neurons carrying the *LRRK2* c.G6055A (p.G2019S) mutation, were significantly mitigated by genomic editing using ABEs or HDR.

### Characterization of mutation-corrected isogenic iPSCs using transcriptomic analysis

**Fig. 5:**
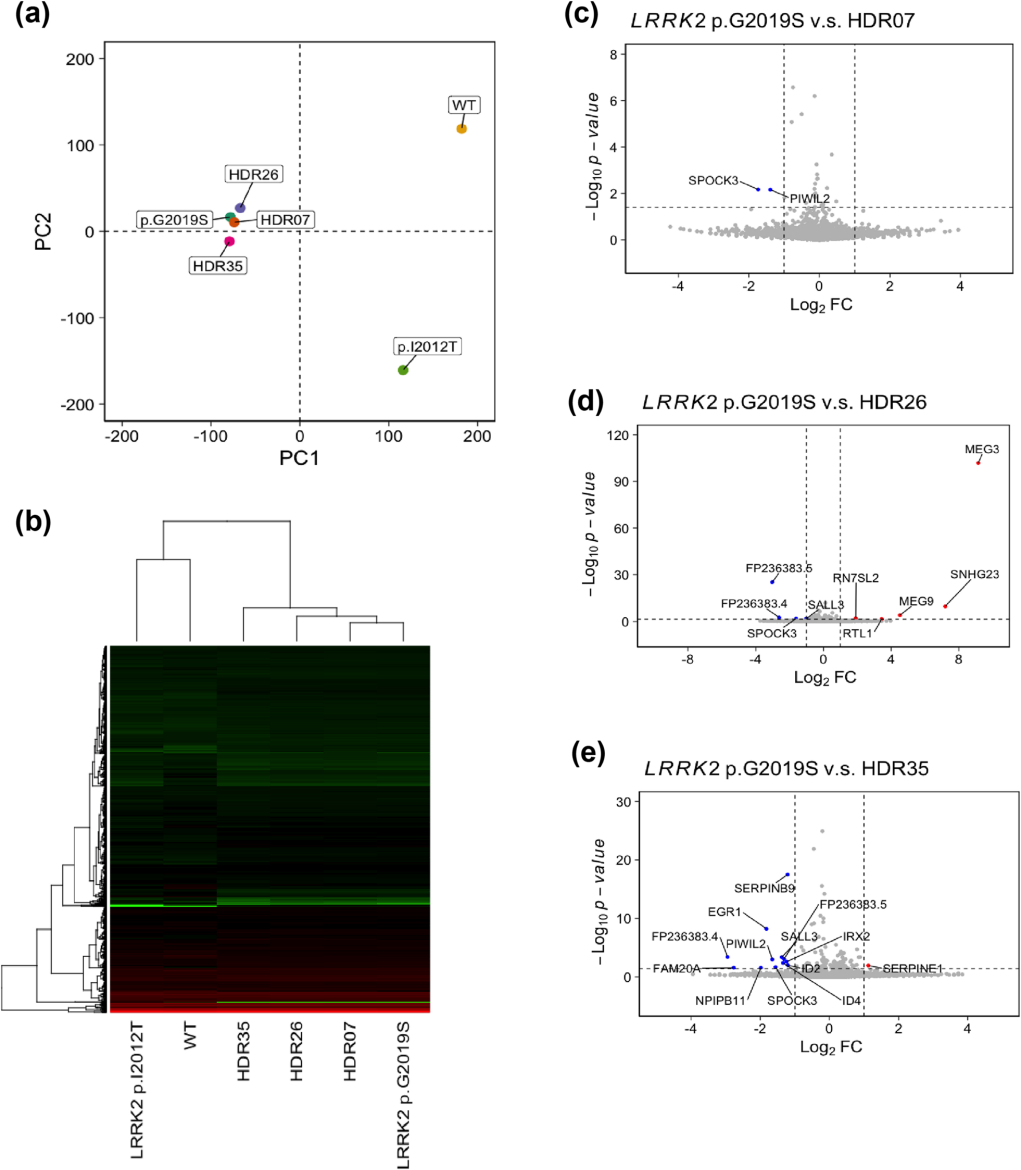
Characterization of isogenic iPSCs corrected for the *LRRK2* p.G2019S mutation using transcriptomic analysis. **a** Principal component analysis (PCA) showing the gene expression profiles of the normal control (wild type, WT), *LRRK2* with p.I2012T, *LRRK2* with p.G2019S, and *LRRK2* with p.G2019S mutation-corrected isogenic iPSC clones (HDR07, HDR26, and HDR35). **b** Heat map of the RNA-Seq transcriptome analysis of 1,547 selected genes from the WT, LRRK2 mutants (G2019S and I2012T), and G2019S-corrected isogenic iPSC clones (HDR07, HDR26, and HDR35). **c** Volcano plot of differentially expressed genes between *LRRK2* p.G2019S and HDR07. **d** Volcano plot of differentially expressed genes between *LRRK2* p.G2019S and HDR26. **e** Volcano plot of differentially expressed genes between *LRRK2* p.G2019S and HDR35

Among the successfully corrected clones from HDR without off-target effects or indels, three independent clones were characterized (Table 1). To examine transcriptome-level differences in iPSCs from a normal control participant, iPSCs derived from a patient carrying the *LRRK2* c.T6035C (p.I2012T) mutation [38], *LRRK2* p.G2019S iPSCs, and the HDR-corrected iPSCs, RNA sequencing analysis (RNA-Seq) was performed. As shown in Fig. 5a, principal component analysis (PCA) showed that the iPSCs from a normal control having wild-type (WT) *LRRK2* genotype, a patient carrying the *LRRK2* c.G6055A (p.G2019S) mutation, and another patient carrying the *LRRK2* c.T6035C (p.I2012T) mutation randomly clustered into three distinct transcriptome groups. Unexpectedly, three mutation-corrected isogenic iPSC clones (HDR07, HDR26, and HDR0735) without off-target editing or indels analyzed by WGS shared a common transcriptome with *LRRK2* p.G2019S iPSCs, but it was distinct from the WT iPSCs, supporting that the isogenic cell lines generated by the gene editing system serve as better control cell lines than iPSCs derived from unrelated healthy control participants. These findings reveal using isogenic cells with mutation correction could reduce the biological variances arising from individuals in different genetic backgrounds. In agreement with the PCA, heat map and hierarchical clustering based on 1,547 selected genes from the WT and mutant iPSCs showed a close correlation between parental *LRRK2* p.G2019S iPSCs and mutation-corrected clone (HDR07, HDR26, and HDR35) iPSCs, rather than the WT control iPSCs derived from an unrelated healthy control individual (Fig. 5b).

To further determine the different gene expression between parental *LRRK2* p.G2019S iPSCs and three isogenic mutation-corrected iPSC lines, more than 17,000 genes were significantly differentially expressed with an absolute fold change ≥ 1.0 for three comparisons: *LRRK2* p.G2019 iPSCs vs. HDR07 iPSCs (Fig. 5c), *LRRK2* p.G2019 iPSCs vs. HDR26 iPSCs (Fig. 5d), and *LRRK2* p.G2019 iPSCs vs. HDR35 iPSCs (Fig. 5e). The expressions varied between *LRRK2* p.G2019S iPSCs and mutation-corrected iPSCs in two genes, *SPOCK3* and *PIWIL2,* which were down-regulated in *LRRK2* p.G2019S iPSCs (Fig. 5c). *SPOCK3* was also identified in the comparison of *LRRK2* p.G2019S iPSCs and HDR26 (Fig. 5d), as well as HDR35 (Fig. 5e).

### Comparative proteomic analysis of mutation-corrected isogenic iPSCs

**Fig. 6:**
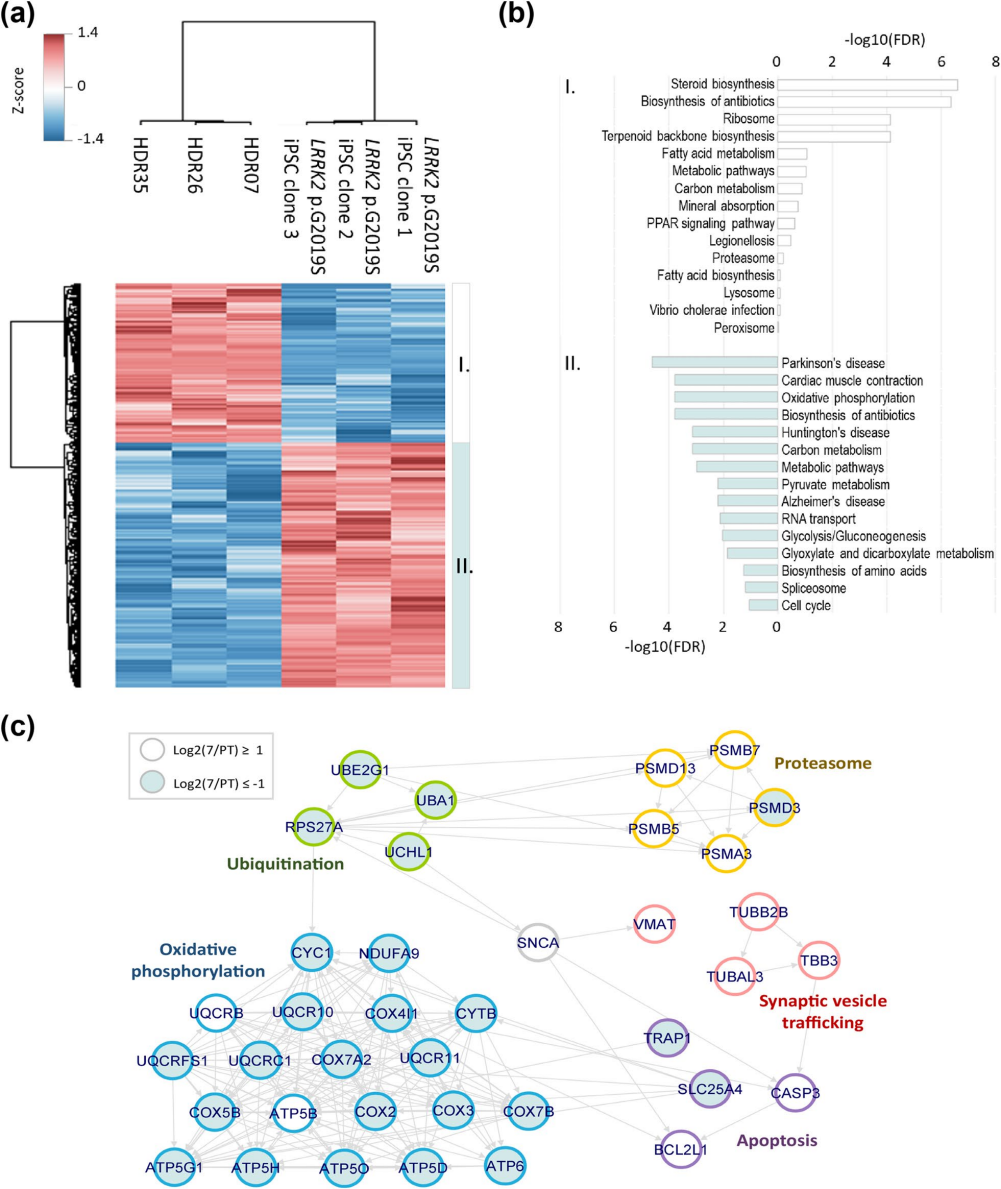
Network analysis of regulated proteins involved in the Parkinson’s disease pathway. **a** Hierarchical clustering of differentially expressed proteins. The color coding shows the relative abundance based on the Z-score transformation. Two different clusters of proteins were subjected to pathway enrichment analysis. **b** The pathway analysis was performed using DAVID Bioinformatics Resource (https://david.ncifcrf.gov) with the Benjamini–Hochberg false discovery rate (FDR) ≤ 0.05 (PMID: 19033363). Empty bar: mutation-corrected isogenic iPSCs; blue bar: *LRRK2* p.G2019S iPSCs. **c** Each circle represents one regulated protein, and different colors represent the distinct sub-pathway inside the PD pathway: green, ubiquitination; yellow, proteasome degradation; blue, oxidative phosphorylation; purple, apoptosis; and pink, synaptic vesicle trafficking. Filled circles indicate that the protein level was increased in *LRRK2 G2019S* iPSCs, whereas down-regulated proteins are indicated as clear circles

Although the gene expression was modestly indistinguishable between *LRRK2* p.G2019S iPSCs and mutation-corrected iPSCs, differentiated neurons exhibited significant alterations in neuropathology, suggesting the presence of an alternative regulatory mechanism for tuning cellular processes after transcription. Supporting this notion, microRNAs (miRNAs), which repress gene expression by regulating the degradation and translation of specific messenger RNAs (mRNAs), have already been implicated in the pathogenesis of PD [39]. To gain more insights into the disease mechanisms in the post-transcriptional level, *LRRK2* p.G2019S iPSCs and corrected HDR07, HDR26, and HDR35 iPSCs, which have a minimal difference in genetic background, were subjected to mass spectrometer (MS)-based label-free quantitative proteomics analysis. A total of 6,469 protein groups were identified at an FDR of 1% at both the peptide and protein levels. Among 5,288 quantified proteins, 583 proteins were significantly up-regulated and 375 proteins significantly down-regulated in *LRRK2* G2019S iPSCs (Fig. 6a).

Pathway enrichment showed that cellular metabolism was apparently different in mutation-corrected isogenic control iPSCs than parental *LRRK2* p.G2019S iPSCs. *LRRK2* p.G2019S iPSCs were prone to use carbohydrates for cellular energy, while the fatty acid metabolism was higher in mutation-corrected iPSCs (Fig. 6b, empty bars). These observations were in line with the clinical studies showing reduced fatty acid metabolism in plasma from patients with PD [40]. In addition, peroxisome proliferator-activated receptor (PPAR) signaling pathway, a lipid sensor [41], was consistently elevated in mutation-corrected iPSCs. The activation of PPAR signaling also increases mitochondrial biogenesis, anti-apoptosis effects, and antioxidant defenses [41]. Furthermore, several PPAR agonists have been shown to exert neuroprotective activity in neurodegenerative disorders, including Alzheimer’s disease (AD), PD, and amyotrophic lateral sclerosis [42, 43]. On the other hand, greater oxidative phosphorylation was found in parental *LRRK2* p.G2019S iPSCs, which may generate excess reactive oxygen species, damaging proteins, and organelles and contributing to PD. As expected, the PD-related pathways were over-represented as the top one pathway in the *LRRK2* p.G2019S iPSCs. Further analysis of proteins involved in PD pathways showed that, in addition to oxidative phosphorylation, ubiquitination was also highly activated in *LRRK2* p.G2019S iPSCs (Fig. 6c). In contrast, proteasome degradation and synaptic vesicle trafficking were suppressed in the *LRRK2* p.G2019S iPSCs.

Taken together, these findings from comparative proteomic analysis suggest that the protein expression profiles of the mutation-corrected isogenic iPSCs were largely different from parental *LRRK2* p.G2019S iPSCs. While increased oxidative phosphorylation and ubiquitination as well as decreased proteasome degradation and synaptic vesicle trafficking pathways were observed in *LRRK2* p.G2019S iPSCs, the elevation of the peroxisome PPAR pathway and fatty acid metabolism was noted in mutation-corrected isogenic iPSCs.

## Discussion

The results of this study demonstrated correction of the most prevalent mutation underlying familial and sporadic PD, *LRRK2* p.G2019S, using CRISPR/Cas9-based HDR and ABEs. In patient-derived iPSCs carrying the *LRRK2* p.G2019S mutation, ABEs efficiently corrected the pathogenic allele with a higher correction rate and lower off-target and indel rate than CRISPR/Cas9-based HDR. The dopaminergic neurons derived from the isogenic clones corrected by ABEs or HDR had a substantial reduction in the abnormally increased LRRK2 kinase activity, α-synuclein expression and phosphorylation, and mitigated neurite degeneration and apoptosis. Comparative transcriptomic and proteomic analysis of the mutant line identified several differentially expressed protein pathways compared to the isogenic controls, including cellular fatty acid metabolism and PPAR signaling, which are potential novel targets relevant to the pathophysiology of PD.

In the current study, HDR-mediated editing generated deletions in 27 of 47 clones (57.4%) and only 3 of 47 clones (6.4%) were on-target edited. These results confirm previous findings that HDR-mediated gene targeting is prone to create off-target editing and indels. Although HDR can be harnessed to insert a specific DNA template for precise correction of the mutation, this mechanism has low efficiency in nondividing cells, e.g., iPSCs and neurons [44–46]. Previous reports demonstrated a 2–5% efficiency of HDR after creating DSBs in iPSCs or embryonic stem cells [47, 48]. On the other hand, the ABEs had a higher on-target correction rate (24.5%) compared to HDR-mediated editing and no indels were detected. The on-target correction rate by ABEs in iPSCs is comparable to its editing efficiency in other cell types, which showed a high on-target editing efficiency (32%-60%) in HEK293T and U2OS cells [18]. The activity window of ABE7.10 is from protospacer position 4 to 7, counting the protospacer-adjacent motif at position 21–23 [18]. It is possible that ABEs act on DNA at off-target loci that contain an adenine base positioned in the activity window of the editor. This off-target editing within the active window occurs in less than 5% of HEK293T cells [18]. However, 11 of 53 clones (20.75%) that had these off-target sites within the active window were identified. Furthermore, 3 clones (5.67%) presented proximal off-target editing, which occurs near the target locus but outside the active window. WGS of the selected clones also demonstrated more distant off-target editing effects. Thus, ABEs result in a more favorable mutation correction rate in iPSCs compared to HDR, without indels, though there is a considerable off-target editing rate.

The gain-of-function *LRRK2* p.G2019S mutation causes abnormal accumulation of α-synuclein [7], impaired neurite outgrowth [8], and increased caspase 3 activity [7, 9] in iPSC-derived dopaminergic neurons. The elevated phosphorylation at Ser1292 of LRRK2, a known marker of active kinase autophosphorylation [36], in dopaminergic neurons derived from *LRRK2* p.G2019S iPSCs was also observed in the current study. The other disease phenotypes, including increased α-synuclein phosphorylation at Ser129 and α-synuclein accumulation, and impaired neurite outgrowth, were also recapitulated by the dopaminergic neurons derived from *LRRK2* p.G2019S iPSCs. Although the expression of TH was not significantly down-regulated in the dopaminergic neurons derived from *LRRK2* p.G2019S iPSCs, the increase in caspase 3 activities further indicates that these neurons were particularly prone to degenerate [49]. ABE- and HDR-mediated genomic correction rescued these disease-related phenotypes in morphology and protein expression in dopaminergic neurons. This validates the potential applicability of genome editing to mitigate the neurodegenerative processes in PD. Further characterization on neurophysiological and functional phenotypes, such as the neuronal firing rate and the number of active channels, will be warranted.

The comparative transcriptomic analysis between *LRRK2* p.G2019S iPSCs and mutation-corrected isogenic controls or iPSCs from an independent healthy participant showed clear separation of the *LRRK2* p.G2019S iPSCs and three isogenic mutation-corrected iPSC clones. Among the genes differentially expressed between *LRRK2* p.G2019S cells and the three isogenic controls, *SPOCK3* and *PIWIL2* were down-regulated in the *LRRK2* p.G2019S iPSCs. *SPOCK3* encodes a protein that participates in inhibiting matrix metalloproteinases (MMPs) involved in degradation of the extracellular matrix. SPOCK3 has also been shown to be up-regulated in astrocytes with aging in AD mice [50]. In addition, the expression of SPOCK3 is significantly enriched in PD mice after L-DOPA treatment [51]. The role of SPOCK3 in relation to *LRRK2* mutation-related PD pathophysiology needs to be explored in future studies. In addition, *PIWIL2* exhibits reduced transcription in *LRRK2* p.G2019S iPSCs. PIWIL2 is involved in the biogenesis of PIWI-interacting RNAs (piRNAs) [52]. A high number of piRNAs are differentially regulated in several cell types derived from patients with PD, including fibroblasts, iPSCs, and cell lines differentiated into midbrain neurons. Notably, previous studies have described deregulated piRNA expression in AD, emphasizing the relevance of piRNAs to the neurodegeneration process [53]. It would be valuable to investigate the role of PIWIL2 in PD, as it may provide relevant novel insights into the epigenetic landscape of PD regulated by small RNAs and elucidate novel disease mechanisms. Further proteomic analyses compared the differentiated dopaminergic neurons from *LRRK2* G2019S iPSCs and corrected-isogenic iPSCs and found that oxidative phosphorylation and ubiquitination are highly activated in the *LRRK2* p.G2019S neurons. In contrast, proteasome degradation and synaptic vesicle trafficking were obviously suppressed in the *LRRK2* p.G2019S cells. Both oxidative phosphorylation and decreased proteasome degradation cause α-synuclein accumulation and promote apoptosis, which are known pathways in PD [54]. In addition, energy metabolism, focusing on fatty acid metabolism, as well as synaptic PPAR pathways is altered in *LRRK2* p.G2019S neurons compared to the isogenic control clones. These results provide further mechanistic insights into identification of potential novel targets relevant to the pathophysiology of PD.

## Conclusions

In summary, the current study showed that ABEs have greater efficiency, lower indel, and off-target rates than CRISPR/Cas9-based HDR in correcting the most prevalent PD mutation, *LRRK2* p.G2019S. Successful correction of this missense gain-of-function mutation mitigated neuropathological hallmarks of PD. Thus, the corrected isogenic cells could provide an ideal control for investigating potential novel targets relevant to the pathophysiology of PD. These results envision that genome editing, especially ABE, could have a promising potential to directly correct the pathogenic mutation in patients with PD in the future.

## Data Availability

The data that support the findings of this study are available from the corresponding author upon reasonable request.

## Abbreviations

CRISPR: Clustered regularly interspaced short palindromic repeat
Cas9: CRISPR-associated protein 9
iPSCs: Induced pluripotent stem cells
LRRK2: Leucine-rich repeat kinase 2
NHEJ: Nonhomologous end joining
HDR: Homology-directed repair
indel: Insertions and deletions
ABEs: Adenine base editors
PAM: Protospacer-adjacent motif
ssODNs: Single-stranded oligodeoxynucleotides
